# Dietary protein sources and risk of diabetic nephropathy in women: A case-control study

**DOI:** 10.1186/s12902-021-00841-3

**Published:** 2021-08-27

**Authors:** Monireh Aziz, Yahya Jalilpiran, Mehdi Nekouimehr, Shaahin Fattahi, Pari Mokhtari, Ahmad Jayedi, Mir Saeed Yekaninejad, Khadijeh Mirzaei

**Affiliations:** 1 Department of Clinical Nutrition, School of Nutrition and Food Sciences, Shiraz University of Medical Sciences, Shiraz, Iran; 2 Department of Clinical Nutrition, School of Nutritional Science and Dietetics, Tehran University of Medical Sciences, Tehran, Iran; 3 Students’ Scientific Research Center (SSRC), Tehran University of Medical Sciences (TUMS), Tehran, Iran; 4 Department of Community Nutrition, School of Nutritional Science and Dietetics, Tehran University of Medical Sciences, Tehran, Iran; 5 Department of Nutrition and Integrative Physiology, College of Health, University of Utah, Salt Lake City, USA; 6 Department of Epidemiology and Biostatistics, School of Public Health, Tehran University of Medical Sciences (TUMS), Tehran, Iran

**Keywords:** Diet, Dietary protein, Diabetic nephropathy, Case-control

## Abstract

**Background:**

Several studies have investigated the association between dietary protein and the risk of diabetic nephropathy (DN); however, there is no agreement on the type of dietary protein sources that might increase the risk of DN. This study was conducted to investigate the associations between different protein sources and the odds of DN developing in Iranian women with existing type 2 diabetes.

**Methods:**

In this case-control study, 105 women with DN and 105 controls, matched for age and diabetes duration, were selected from the Kowsar Diabetes Clinic in Semnan, Iran. Dietary intake was assessed using a validated and reliable food frequency questionnaire. Dietary protein patterns were estimated using the factor analysis method. Multivariate logistic regression was performed to examine the association between protein patterns and the odds of developing DN.

**Results:**

Two patterns were identified: the Mediterranean-based Dietary Protein Sources (MDPS) pattern which is rich in low-fat dairy, fish, poultry, soy, and legumes, and the Western-based Dietary Protein Sources (WDPS) pattern, rich in red and processed meats, eggs, and high-fat dairy. After adjusting for several confounders, greater adherence (third vs. the first tertile) to the MDPS pattern was associated with lower odds of DN (OR = 0.03; 95 % CI: 0.00, 0.10). In contrast, a strong positive association was observed between adherence to the WDPS pattern and DN (OR = 2.81; 95 % CI: 1.09–7.21).

**Conclusions:**

Our results show that there is a potential association between the type of protein sources consumed and the odds of DN development in women with type 2 diabetes. Further studies are needed to confirm these findings.

## Introduction

Diabetic kidney diseases affect about 40 % of patients with type 2 diabetes (T2D) [1]. Long-term hyperglycemia in diabetic patients causes disorders in various organs, including the kidney. The severity of this damage is assessed by the glomerular filtration rate (GFR) and proteinuria [2]. A urinary protein level of more than 300 mg/day in diabetic patients is an indicator for the diagnosis of diabetic nephropathy (DN) [3]. DN incidence is reported to be about 3 % per year, and the disease occurs about 10 to 20 years after the onset of T2D [4]. According to a recent systematic review, the prevalence of DN among Iranian adults ranges from 7 to 26 % [5]. Family history, gestational diabetes mellitus, hypertension, lipid profile disorders, obesity, insulin resistance, and elevated glycosylated hemoglobin levels are some risk factors for DN [6]. An important approach in the prevention of diabetic kidney disease is to maintain kidney function through a balanced dietary pattern and drug therapy [7].

With regard to dietary factors, previous studies showed moderate beneficial effects with low-protein diets, improving renal function in patients with DN [8]. These results are based on the hypothesis that protein-rich diets increase glomerular blood pressure, both byactivating the renin-angiotensin system and by promoting disease progression in damaged kidneys [9]. In contrast, some studies did not approve the beneficial effect of low-protein diets in improvement of renal function in chronic kidney diseases [10–12]. In a cross-sectional study, a dietary pattern rich in vegetables and fish was significantly correlated with lower serum creatinine and higher estimated GFR [13]. Moreover, previous studies have shown that a diet without any red meat, replacing it with chicken meat, reduced urinary albumin secretion [14, 15]. There is evidence that plant-based dietary proteins may improve kidney function in patients with T2D [16, 17].

In general, there is no agreement on the amounts and types of ditary protein to recommend in patients with kidney diseases. Recent epidemiological studies have only focused on associations between dietary patterns and –disease, instead of individual foods and nutrients related to chronic diseases [18]. Besides, the aims of the studies have changed from examining the amount of protein to the type of protein intake in kidney diseases [16]. Therefore, this case-control study was conducted to address these gaps by examining the association between dietary protein sources and the risk of diabetic nephropathy in women with DN.

## Methods

### Participants

In this case-control study, participants were recruited from the Kowsar Diabetes Clinic in Semnan, Iran, from July to December 2016. Patients were eligible for enrollment in this study if they were women with prevalent T2D, aged between 30 and 65 years, and with a history of 3–10 years of T2D. The definition of diabetes used in this study is based on the American Diabetes Association’s new diagnostic criteria: fasting blood glucose (FBG) ≥ 126 mg/dl, or 2-hour post-load blood glucose (2hrBG) ≥ 200 mg/dl; glycosylated hemoglobin (HbA1c) ≥ 6.5 % [19]. Participants were not included if they had autoimmune disorders or previous history of cancer, coronary angiography, hepatic disease, myocardial infarction, or stroke. Total energy intake of < 500 or > 3500 kcal/day and/or poor response to the food-frequency questionnaire (FFQ) were considered to be exclusion criteria.

In this study, DN is defined as urinary albumin-to-creatinine ratio (ACR) ≥ 30 mg/g in a random spot urine sample [20]. In total, 120 patients with DN were identified through convenience sampling. 105 patients agreed to participate in the study. 105 diabetic women without DN were selected as the control group from the same center by a 1:1 matching to the DN cases, by age at 1-year intervals and by the duration of diabetes in 6-months intervals. All participants provided written informed consent to participate in our study.

### General data collection

Participants’ data including age, diabetes duration, and smoking status were recorded, while weight (kg) was measured while subjects were wearing light clothing without shoes. Body mass index (BMI, kg/m^2^) was calculated as weight in kilograms divided by the square of height in meters. Systolic and diastolic blood pressure was measured once on the left arm while sitting after a resting period ≥ 5 min using a manual sphygmomanometer. A standard physical activity questionnaire (IPAQ) [21] was used to assess individuals’ physical activity. Scoring criteria based on this questionnaire indicated “low physical activity” (score < 600 Metabolic Equivalent of Task-hours/week), “moderate physical activity” (score 600–3000 MET-h/week) or “high physical activity” (score > 3000 METh/week) levels.

### Examination of blood biomarkers

Biochemical variables including FBG, 2hrBG, HbA1c, triglycerides (TG), low-density lipoprotein (LDL), total cholesterol (TC), high-density lipoprotein (HDL), total serum creatinine (Cr), and blood urea nitrogen (BUN) were obtained from participants’ medical records from the previous three months.

### Dietary intake assessment

Dietary intake was assessed using a validated and reliable food-frequency questionnaire (FFQ) through face-to-face interviews [22]. Participants reported their intake of food or food items daily, weekly, monthly, or yearly. Final portion sizes were converted into g/day using household measurements. Then, these amounts were adjusted for energy intake using the residual method [23]. To estimate energy and nutrient intakes, dietary intakes were analyzed using NUTRITIONIST 4 (First Data Bank, San Bruno, CA) software.

### Statistical analysis

Initially, dietary protein sources were categorized into eight groups based on similarity in their nutrients and/or culinary usage (low-fat dairy, high-fat dairy, poultry, legumes, soy, fish, and red and processed meats). Then, principal component analysis (PCA) was performed on these eight categories of dietary protein sources, considering two factors with eigenvalues > 2, a rotated factor loading greater than 0.3. Factor loadings correspond to the strength of the correlation coefficients between dietary protein source patterns and dietary protein subtypes. A negative loading value reveals an inverse relationship, and a positive loading value indicates a positive association. The normal distribution of the quantitative variables was assessed using the Kolmogorov-Smirnov test. Quantitative variables including age, BMI, and diabetic duration were compared between cases and controls using the paired-samples t-test. One-way ANOVA and chi-square tests were used to compare quantitative variables across the tertiles of dietary protein source patterns and to determine the distribution of the qualitative variables across the tertiles of dietary protein source patterns, respectively. Energy-adjusted dietary macro and micronutrient intakes, across the tertiles of dietary protein source patterns, were compared using analysis of covariance (ANCOVA). Conditional logistic regression for matched analysis was used to determine whether different dietary protein sources are associated with the risk of DN. In adjusted models, age, body mass index, energy intake, physical activity, diabetes duration, cardiovascular disease history, and type of drug used (angiotensin receptor blockers, angiotensin-converting enzyme inhibitors, beta-blockers, metformin, sulphonylurea, and insulin) were controlled. The Mantel-Haenszel extension chi-square test was used to assess the overall trend of the odds ratio across increasing tetiles of dietary protein pattern scores. Data analysis was performed using SPSS software (Version 25, SPSS Inc., Chicago, IL, USA) and *P* < 0.05 was considered statistically significant.

## Results

Factor loadings of dietary protein sources contributing to two identified dietary protein source patterns are shown in Table 1. Two major patterns were identified: the Mediterranean-based Dietary Protein Sources (MDPS) pattern, rich in low-fat dairy, fish, poultry, soy, and legumes; and the Western-based Dietary Protein Sources (WDPS) pattern, rich in red and processed meats, eggs, and high-fat dairy. Generally, these patterns accounted for 50 % of the variance in the food groups.

**Table 1:** Food groups used in the factor analysis and factor loadings for each dietary pattern among 105 cases and 105 controls

Food groups	Food items	Mediterranean-based dietary protein sources pattern	Western-based dietary protein sources pattern
Low-fat dairy	Low-fat and flavored milk, low-fat yogurt, cheese, kashk, and doogh	0.839	
Poultry	Chicken, turkey, ostrich	0.730	
Legumes	Lentil, beans, lentils, and peas	0.570	
Soy	Soy and soy products	0.480	
Fish	All fish types	0.460	
Egg	Egg		0.799
Red and processed meats	Beef and veal, sheep, lamb, minced meat, hamburger, sausages, Liver, kidney, heart, offal, rennet, tongue, and brain		0.752
High-fat dairy	High-fat milk and yogurt, ice-cream, cream, and creamy cheese	-0.559	0.652
Explained variance (%)	----	29.81	20.52

Sociodemographic characteristics and anthropometric measures of study participants are presented in Table 2. The results show that the usage of angiotensin receptor blockers (*P* = 0.04) and angiotensin-converting enzyme inhibitors (*P* = 0.001) were more in DN cases than in controls. Other characteristics were not found to be different between the cases and the controls (*P* > 0.05).

**Table 2:** Sociodemographic characteristics and anthropometric measures of study participants

*Variables*	*Total(N = 210)*	*Cases(N = 105)*	*Controls(N = 105)*	*p (paired t-test)*
Age (year)	55.37 (7.0)	55.3 (7.0)	55.4 (7.1)	0.94
Body mass index (kg/m2)	28.10 (4.6)	28.7 (4.7)	27.5 (4.4)	0.06
Diabetes duration (years)	7.58 (2.2)	7.6 (2.2)	7.6 (1.1)	0.88
Physical activity				0.13
Low	68 (32.4)	31 (29.5)	37(35.2)	
Moderate	40 (33.3)	42 (40.0)	28 (26.7)	
High	72 (34.3)	32 (30.5)	40(38.1)	
History of cardiovascular disease (yes)	47 (22.4)	24 (22.9)	23 (21.9)	1.00
Medications usage (yes)				
Angiotensin receptor blockers	105 (50.0)	60 (57.1)	45 (42.9)	0.05
Angiotensin converting enzyme inhibitors	65 (31.0)	44(41.9)	21(20.0)	0.001
Beta blockers	38 (18.1)	20 (19.0)	18 (17.1)	0.72
Metformin	208 (99.0)	104 (99.0)	104 (99.0)	1.00
Sulfonylureas	133 (63.3)	71(67.6)	62 (59.0)	0.25
Insulin	61 (29)	26 (24.8)	35 (33.3)	0.22

A) Data are presented as mean (standard deviation) or Number (%).

B) Chi-square test or Fisher’s exact test were used for comparison of qualitative variables.

General characteristics of participants across tertiles of dietary protein source patterns ar presented in Table 3. The results show that ACR (*p* < 0.001), serum albumin (*p* = 0.001), FBS (*p* = 0.005), serum HbA1c (*p* < 0.001), serum TC (*p* = 0.005), serum LDL cholesterol (*p* = 0.004), serum creatinine (*p* = 0.01), and BUN (*p* = 0.003) were significantly decreased across the tertiles of the MDPS pattern score. Greater adherence to the WDPS pattern was associated with increased ACR (*p* = 0.02), FBS (*p* = 0.03), and usage of angiotensin-converting enzyme inhibitors (*p* = 0.003). Greater aderence to the Western pattern was also associated with decreased serum TG (*p* = 0.001).

**Table 3:** General characteristics and biochemical markers of participants across tertiles of dietary protein sources patterns among 105 cases and 105 controls^a^

Variable	Mediterranean-based dietary protein sources pattern				Western-based dietary protein sources pattern			
	**Tertile 1(< -0.72)**	**Tertile 2(-0.72–0.60)**	**Tertile 3(> 0.60)**	**P trend**^**b**^	**Tertile 1(< -0.36)**	**Tertile 2(-0.36–0.18)**	**Tertile 3(> 0.18)**	**P trend**
Age (y)	55.17 ± 6.94	55.03 ± 7.51	55.91 ± 6.818	0.54	55.73 ± 6.44	55.26 ± 7.18	55.13 ± 7.63	0.62
BMI (kg/m2)	28.99 ± 5.25	27.04 ± 4.46	28.26 ± 3.81	0.34	28.72 ± 4.17	28.25 ± 4.67	27.32 ± 4.87	0.07
Diabetes duration (y)	7.38 ± 2.1	7.88 ± 2.18	7.48 ± 2.26	0.80	7.61 ± 2.24	7.69 ± 2.13	7.44 ± 2.19	0.64
ACR	214.41 ± 128.62	102.08 ± 123.84	59.78 ± 97.55	< 0.001	96.04 ± 122.65	132.24 ± 142.7	147.99 ± 132.31	0.02
Albumin (mg/dl)	13.71 ± 7.47	12.62 ± 13.03	7.83 ± 8.12	0.001	10.09 ± 8.81	12.17 ± 13.44	11.9 ± 7.11	0.29
SBP (mmHg)	123.37 ± 16.5	123.19 ± 17.81	136.89 ± 120.318	0.26	121.76 ± 15.68	135 ± 120.98	126.69 ± 14.78	0.68
DBP (mmHg)	82.36 ± 13.64	80.76 ± 13.22	81.23 ± 10.47	0.60	82.9 ± 12.59	79.66 ± 13.41	81.79 ± 11.32	0.60
FBS (mg/dl)	171.36 ± 52.68	162.06 ± 50.19	148.53 ± 38.52	0.005	155.81 ± 56.96	152.5 ± 37.59	173.63 ± 56.26	0.03
HB A1c (%)	8.73 ± 1.39	8.44 ± 1.39	7.87 ± 1.26	< 0.001	8.28 ± 1.3	8.04 ± 1.35	8.71 ± 1.44	0.06
TC (mg/dl)	187.96 ± 37.18	181.74 ± 35.41	171.1 ± 32.6	0.005	175.53 ± 37.75	182.79 ± 35.83	182.49 ± 33.21	0.25
TG (mg/dl)	174.8 ± 56.71	161.4 ± 63.66	158.06 ± 64.41	0.11	180.97 ± 76.07	166.1 ± 54.89	147.19 ± 46.86	0.001
LDL (mg/dl)	106.43 ± 30.74	104.54 ± 33.8	91.21 ± 26.79	0.004	96.53 ± 30.7	104.49 ± 33.14	101.17 ± 29.52	0.40
HDL (mg/dl)	44.79 ± 9.27	46.46 ± 9.77	45.89 ± 8.76	0.48	46.11 ± 9.1	45.94 ± 9.12	45.07 ± 9.64	0.51
Creatinine (mg/dl)	0.93 ± 0.16	0.91 ± 0.17	0.86 ± 0.17	0.01	0.89 ± 0.16	0.91 ± 0.18	0.89 ± 0.17	0.77
BUN (mg/dl)	16.52 ± 4.97	15.54 ± 3.61	14.39 ± 3.73	0.003	15.7 ± 3.63	15.48 ± 4.93	15.27 ± 4.04	0.55
PA (%)								
Low	19(27.1)	25(35.7)	24(34.3)	0.68^c^	28(40)	19(27.1)	21(30)	0.44
Moderate	27(38.6)	23(32.9)	20(28.6)		23(32.9)	23(32.9)	24(34.3)	
High	24(34.4)	22(31.4)	26(37.1)		19(27.1)	28(40.0)	25(35.7)	
CVD history (%)	15(21.4)	18(25.7)	14(20)	0.7	11(15.7)	15(21.4)	21(30)	0.12
ARB drugs user (%)	34(48.6)	39(55.7)	32(45.7)	0.48	32(45.7)	33(47.1)	40(57.1)	0.34
ACEI drugs user (%)	23(32.9)	27(38.6)	15(21.4)	0.08	13(18.6)	20(28.6)	32(45.7)	0.002
Beta-blocker drugs user (%)	12(17.1)	15(21.4)	11(15.7)	0.59	11(15.7)	10(14.3)	17(24.3)	0.32
Metformin user (%)	70(100)	68(97.1)	70(100)	0.13	69(98.6)	69(98.6)	70(100)	0.60
Sulfonylurea drugs user (%)	47(67.1)	45(64.3)	41(58.6)	0.56	43(61.4)	47(67.1)	43(61.4)	0.72
Insulin user (%)	18(25.7)	23(32.9)	20(28.6)	0.64	21(30.0)	17(24.3)	23(32.9)	0.52

Abbreviations: BMI, body mass index; ACR, albumin creatinine ratio; SBP, systolic blood pressure; DBP, diastolic blood pressure; FBS, fasting blood sugar; HB, hemoglobin; TC, total cholesterol; TG, triglyceride; LDL, low density lipoprotein; HDL, high density lipoprotein; blood urine nitrogen; PA, physical activity; CVD, cardiovascular disease; ARB, angiotensin receptor blockers; ACIE, angiotensin converting enzyme inhibitors.

^a^ Data are presented as mean ± SD or number(percent).

^b^ Anova test was used.

^c^ Chi−square test was used.

Energy-adjusted dietary intakelevels across tertiles of identified dietary protein sources patterns are shown in Table 4. Increased adherence to the MDPS pattern was associated with increased intake of protein (*p* = 0.001), carbohydrates (*p* = 0.001), cholesterol (*p* = 0.001), folate (*p* = 0.001), and vitamin B_12_ (*p* = 0.001). Also, higher adherence to this pattern was associated with decreased intake of vitamin B_6_ (*p* = 0.01), calcium (*p* = 0.005), sodium (*p* = 0.03), and magnesium (*p* = 0.02). Greater adherence to the WDPS pattern was associated with an increased intake of cholesterol (*p* < 0.001) and vitamin B_12_ (*p* < 0.001). Moreover, increased adherence to this pattern was associated with decreased intake of energy (*p* < 0.001), vitamin A (*p* = 0.008), vitamin K (*p* = 0.03), vitamin E (*p* < 0.001), vitamin C (*p* = 0.04), vitamin B9 (*p* = 0.04), potassium (*p* < 0.001), iron (*p* = 0.01), and magnesium (*p* < 0.001).

**Table 4:** Dietary intakes of participants across tertiles of dietary protein patterns among 105 cases and 105 controls^a^

Variables	Mediterranean-based dietary protein sources pattern				Western-based dietary protein sources pattern			
	Tertile 1(< -0.72) (*N* = 70)	Tertile 2(-0.72–0.60) (*N* = 70)	Tertile 3(> 0.60) (*N* = 70)	**P-value**^**b**^	**Tertile 1**(< -0.36) (*N* = 70)	Tertile 2(-0.36–0.18) (*N* = 70)	Tertile 3(> 0.18) (*N* = 70)	**P-value**
Energy (kcal/day) ^c^	1420.94 ± 192.58	1442.79 ± 278.98	1426.14 ± 373.14	0.90	1498.72 ± 338.58	1304.89 ± 173.61	1486.26 ± 292.84	< 0.001
Protein (gr/day)	45.04 ± 5.93	47.85 ± 8.29	48.08 ± 12.15	0.001	47.48 ± 0.57	46.38 ± 0.58	47.11 ± 0.57	0.41
Carbohydrate (gr/day)	249.04 ± 32.2	252.78 ± 50.36	252.08 ± 77.27	0.001	252.79 ± 1.82	251.32 ± 1.86	249.79 ± 1.81	0.50
Total fat (gr/day)	33.98 ± 7.99	33.21 ± 8.49	31.73 ± 7.43	0.05	32.61 ± 0.7	33.37 ± 0.71	32.94 ± 0.69	0.76
Cholesterol (mg/day)	4.7 ± 3.4	6.79 ± 7.61	8.72 ± 11.23	0.004	5.24 ± 0.82	5.41 ± 0.84	9.56 ± 0.82	< 0.001
Saturated fat (gr/day)	6.38 ± 1.58	6.21 ± 1.61	6.04 ± 1.76	0.84	6.26 ± 0.14	6.17 ± 0.14	6.2 ± 0.14	0.90
Vitamin A (RAE/day)	39.29 ± 18.33	42.6 ± 19.41	37.02 ± 17.12	0.20	44.76 ± 2.05	35.81 ± 2.1	38.34 ± 2.04	0.008
Vitamin K(µg/day)	13.38 ± 5.53	13.53 ± 5.09	12.35 ± 4.28	0.25	14.12 ± 0.52	12.98 ± 0.53	12.16 ± 0.52	0.03
Vitamin E (mg/day)	4.07 ± 0.84	3.91 ± 1.29	4.23 ± 2.05	0.10	4.39 ± 0.13	4.23 ± 0.13	3.59 ± 0.13	< 0.001
Vitamin C (mg/day)	10.36 ± 4.23	10.87 ± 6.7	9.98 ± 6.14	0.70	10.25 ± 0.64	9.31 ± 0.66	11.65 ± 0.64	0.04
Vitamin B1 (mg/day)	1.68 ± 0.22	1.71 ± 0.31	1.67 ± 0.43	0.79	1.68 ± 0.03	1.69 ± 0.03	1.7 ± 0.03	0.84
Vitamin B2 (mg/day)	0.97 ± 0.15	0.99 ± 0.17	0.95 ± 0.24	0.36	0.95 ± 0.02	0.97 ± 0.02	0.99 ± 0.02	0.21
Vitamin B3 (mg/day)	16.34 ± 2.37	16.09 ± 2.84	15.76 ± 3.68	0.13	15.82 ± 0.23	16.19 ± 0.23	16.19 ± 0.23	0.41
Vitamin B5 (mg/day)	2.44 ± 0.74	2.43 ± 0.57	2.5 ± 0.67	0.65	2.4 ± 0.07	2.45 ± 0.07	2.52 ± 0.07	0.44
Vitamin B6 (mg/day)	0.79 ± 0.11	0.78 ± 0.15	0.73 ± 0.14	0.01	0.78 ± 0.02	0.78 ± 0.02	0.75 ± 0.02	0.19
Vitamin B9 (µg/day)	360.57 ± 57.53	394.3 ± 82.12	397.15 ± 129.72	0.01	399.76 ± 8.77	367 ± 8.98	385.26 ± 8.74	0.04
Vitamin B12 (µg/day)	0.12 ± 0.07	0.15 ± 0.13	0.17 ± 0.2	0.04	0.12 ± 0.02	0.12 ± 0.02	0.2 ± 0.02	< 0.001
Sodium (mg/day)	3705.81 ± 1098.67	3584.09 ± 913.78	3285.36 ± 943.92	0.03	3453.85 ± 117.95	3520.6 ± 120.69	3600.81 ± 117.55	0.67
Potassium (mg/day)	1689.10 ± 223.39	1668.51 ± 311.38	1765.76 ± 556.14	0.09	1859.33 ± 33.86	1677.14 ± 34.65	1586.91 ± 33.75	< 0.001
Calcium (mg/day)	406.16 ± 67.32	415.64 ± 65.02	389.78 ± 83.62	0.005	400.52 ± 5.25	399.36 ± 5.37	411.7 ± 5.23	0.19
Iron (mg/day)	14.85 ± 1.79	15.13 ± 2.47	14.75 ± 2.84	0.41	15.17 ± 0.14	14.97 ± 0.14	14.56 ± 0.14	0.01
Phosphorous (mg/day)	911.51 ± 121.28	913.87 ± 176.08	895.16 ± 189.35	0.63	925.53 ± 13.7	913.42 ± 14.02	881.59 ± 13.66	0.06
Magnesium (mg/day)	355.64 ± 44.61	356.93 ± 74.84	354.46 ± 97.47	0.02	380.28 ± 7.1	359.58 ± 7.27	327.16 ± 7.08	< 0.001
Zinc (mg/day)	7.89 ± 1.13	8.29 ± 1.79	8.38 ± 3.29	0.34	8.62 ± 0.23	8.27 ± 0.23	7.68 ± 0.23	0.12

^a^ Data are presented as mean ± SE (except of energy intake that presented as mean ± SD)

^b^ Ancova test was used

^c^ Anova test was used

Crude and multivariable odds ratios (OR) and 95 % confidence intervals (CI) of DN by tertiles of dietary protein source pattern are shown in Table 5. After adjusting for potential confounders (age, body mass index, energy intake, physical activity, diabetes duration, cardiovascular disease history, and drug usage (angiotensin receptor blockers, angiotensin-converting enzyme inhibitors, beta-blockers, metformin, sulphonylurea, and insulin)), presence in the third tertile as opposed to the first tertile of the MDPS pattern was associated with lower odds of having DN (OR = 0.03; 95 % CI: 0.00–0.10). In contrast, greater adherence to the WDPS diet (third vs. the first tertile) was associated with increased odds of disease after adjustment for the aforementioned potential confounders (OR = 2.81; 95 % CI: 1.09–7.21).

**Table 5:** Odds ratios and 95 % confidence intervals of diabetic nephropathy according to tertiles of dietary pattern intake among 105 cases and 105 controls^a^

Patterns	Categories of dietary protein sources patterns			
	**Tertile 1**	**Tertile 2**	**Tertile 3**	**P trend**
Mediterranean-based dietary protein sources pattern	(< -0.72)	(-0.72–0.60)	(> 0.60)	
No. cases/controls	60/10	27/43	18/52	
Crude	1.00 (Ref )	0.13(0.05–0.34)	0.06(0.02–0.17)	< 0.001
Model 1	1.00 (Ref )	0.13(0.05–0.36)	0.05(0.01–0.16)	< 0.001
Model 2	1.00 (Ref )	0.06(0.02–0.25)	0.03(0.00-0.10)	< 0.001
Western-based dietary protein sources pattern	(< -0.36)	(-0.36–0.18)	(> 0.18)	
No. cases/controls	26/44	38/32	41/29	
Crude	1.00 (Ref )	2.00(1.01–3.97)	2.45(1.20-5.00)	0.01
Model 1	1.00 (Ref )	2.26(1.04–4.90)	3.03(1.38–6.69)	0.01
Model 2	1.00 (Ref )	2.20(0.93–5.22)	2.81(1.09–7.21)	0.03

^a^ Conditional logistic regression was used.

Data are presented as odds ratio (95 % confidence interval).

Model 1: Adjusted for age, body mass index, energy intake, and physical activity.

Model 2: Adjusted for confounders in model 1 plus diabetes duration, cardiovascular diseases history, and drug usage (angiotensin receptor blockers; angiotensin converting enzyme inhibitors, beta-blockers, metformin, sulphonyl urea, and insulin).

## Discussion

In this case-control study, we found a significant inverse association between greater adherence to the MDPS diet and odds of having DN. In contrast, there was a positive direct relationship between the WDPS dietand the likelihood of developing DN. It is of great interest that the results of this study do highlight the impact and role of types of dietary protein intake on the odds of having DN.

Our results extend previous studies showing the beneficial effects of Mediterranean-based dietary protein components on kidney function. For example, in one nested case-control study, a higher intake of fish protein (9.35 gr/day vs. 2.72 gr/day), independent of fish fats, was associated with a lower risk of microalbuminuria among young Swedish patients with type 1 diabetes [24]. Similarly, in a cross-sectional study of Greek adolescents, a higher Mediterranean diet Quality Index score was associated with lower levels of albuminuria [25]. Replacing red meat with a chicken-based diet improved urinary albumin excretion rates and lipid profiles in patients with T2D and with microalbuminuria in the short-term [14, 15], and reduced urinary albumin excretion rates long-term [26]. A randomized crossover clinical trial investigated the effect of a normal protein diet (substituting poultry and fish with red meat), compared to a low protein diet, on glomerular hyperfiltration in normoalbuminuric insulin-dependent diabetes mellitus patients. The results of this study showed similar beneficial effects of both diets on GFR [27]. Intervention with 200 mL/day of probiotic soymilk (a soy-based product) in type 2 diabetic patients improved indexes of kidney function (albuminuria, serum creatinine, and estimated glomerular filtration rate) after two months’ intervention [28]. Moreover, after reviewing some cohort studies, researchers concluded that dairy consumption had protective effects on GFR [29]. However, these findings were based on total reported dairy consumption, and further investigation is needed to specify the effects of dairy product subtypes on kidney function.

According to our study, greater adherence to the WDPS diet is associated with an increased likelihood of DN. It seems that diets high in animal protein and with low intake of fruits, vegetables, and fiber may detrimentally result in kidney disease [30]. In addition, a vegetarian diet showed lower serum phosphorous, and decreased fibroblast growth factor levels by 23 times, compared to meat diets, highlighting the fact that the source of protein has a significant effect on phosphorus homeostasis in chronic kidney disease (CKD) patients [31]. Moreover, the replacement of red meat with chicken reduced urinary albumin secretion [14, 15].

In general, several mechanisms might be involved in the relationship between dietary protein sources and DN. First, an increased risk of DN following greater adherence to the WDPS pattern might be due to the amino acid composition of the components of this pattern. Recent research has focused on the association between the accumulation of uremic toxins due to gut dysbiosis and risk of cardiovascular disease (CVD) among patients with CKD [32, 33]. These studies conclude that high consumption of red meats, dairy products, and eggs, which are rich dietary sources of choline and L-carnitine, increases the production of toxins such as p-cresyl sulfate, trimethylamine n-oxide, indoxyl sulfate, and indole-3-acetic acid [34]. These toxins are associated with higher levels of inflammatory markers in patients with CKD [35]. It is also suggested that indoxyl sulfate is associated with endothelial dysfunction, oxidative stress, and monocyte activation [36]. And several studies show potential associations between uremic toxins and mortality due to CKD, CVD, and kidney disease progression [37–40]. Second, the inverse association between the MDPS pattern and the odds of developing DN might be due to the high fiber content of this pattern, whichincludes legumes and soy, plant-based proteins with a high amount of fiber [41]. These special sources of protein lead to a lower protein-fiber ratio, which substantially correlates with lower levels of indoxyl sulfate and p-cresyl sulfate in CKD patients [42]. Third, the MDPS pattern includes soy and soy products, whose beneficial effects in prevention of kidney disease have been previously reported when, dietary low-fat soy milk powder in an experimental study suppressed myofibroblast differentiation, renal injury, and renal macrophage infiltration -- and therefore prevented DN in diabetic patients [43].

This study has multiple contributions. First, we assessed the association between major protein sources and the odds of developing DN for the first time. Second, all the cases and controls were selected from the same location, during the same period. Third, dietary intakes were assessed using a validated and reliable FFQ. However, we acknowledge some limitations in our research method. First, due to the case-control design of the study, the potential for recall and selection biases must be considered. Second, although we matched cases and controls based on age and diabetes duration, other related factors such as BMI were not considered. Third, the sample size we used in this study is relatively small and there is a need for further research with a larger sample size. Fourth, despite our adjustment for some confounding factors, residual confounding bias cannot be ruled out.

To date, several studies have investigated the impact of individual protein sources on the progression of kidney diseases. However, the results were inconsistent and there was no study investigating the associations between the pattern of protein intake and the risk of such disease. The results of this case-control study show an inverse association between greater adherence to a dietary pattern rich in Mediterranean-style dietary protein sources such as fish, legumes, soy, and low-fat dairy products and the odds of nephropathy in women with T2D. A strong positive association with DN is observed between higher adherence to a dietary pattern rich in Western-style dietary protein sources such as high-fat dairy products, egg, and red and processed meats. Further studies with larger sample sizes are needed to confirm these findings.

## Data Availability

The datasets generated and/or analyzed during the current study are not publicly available as per the rules and regulations of the Community Nutrition Department of Tehran University of Medical Science, but are available from the corresponding author upon request.
